# NATURALLY OCCURRING VARIABILITY IN FINANCIAL STRESS AND INFLAMMATORY BIOMARKERS AMONG OLDER ADULTS

**DOI:** 10.1093/geroni/igz038.015

**Published:** 2019-11-08

**Authors:** Laura Samuel, Sarah L Szanton, Neal Fedarko, Eleanor M Simonsick

**Affiliations:** 1 Johns Hopkins University School of Nursing, Baltimore, Maryland, United States; 2 Johns Hopkins School of Medicine, Baltimore, Maryland, United States; 3 Longitudinal Studies Section, Intramural Research Program, National Institute on Aging, Baltimore, Maryland, United States

## Abstract

Financial strain (i.e. difficulty making ends meet) is associated with earlier disability and mortality among older adults, but inflammatory response to financial stress is untested. This study leverages monthly increases in financial stress among older adults receiving Social Security payments monthly on the 3rd. The Health ABC Study randomly sampled black and white adults aged 70-79 free of disability from Memphis, TN and Pittsburgh, PA in 1997 and 1998. Baseline biomarkers included TNF α, IL-6, CRP, and soluble receptors were measured for about half the sample (IL-2, IL-6, and TNF α Type I and II). Days since Social Security payment was calculated using clinic visit date, which naturally varied in the sample. Restricted cubic spline models stratified by financial strain status tested hypothesized associations, adjusting for financial strain scores, food insecurity, employment status and monthly invariant potential confounders (age, gender, number of inflammatory conditions, waist circumference and anti-inflammatory medications). Among those with financial strain, each additional day since payment was associated with higher TNF-α levels during first week of the month (n=1,633, coefficient=0.0830, p=0.030) and higher IL-6 soluble receptor levels during the last week of the month (n=828, coefficient=12033.44, p=0.037), but not with other biomarkers nor during the middle of the month. Days since payment was not associated with biomarkers among those without financial strain. These results suggest upregulation of pro-inflammatory and not anti-inflammatory pathways during the beginning and end of the month among financially strained older adults. Financial strain is modifiable and these results call attention to addressing it.

